# Continuous expansion of the geographic range linked to realized niche expansion in the invasive Mourning gecko *Lepidodactylus lugubris* (Duméril & Bibron, 1836)

**DOI:** 10.1371/journal.pone.0235060

**Published:** 2020-07-06

**Authors:** Dario Nania, Morris Flecks, Dennis Rödder

**Affiliations:** 1 Zoologisches Forschungsmuseum Alexander Koenig, Bonn, Germany; 2 Department of Biology and Biotechnology, Sapienza University of Rome, Rome, Italy; University of Delhi Department of Environmental Studies, INDIA

## Abstract

*Lepidodactylus lugubris* is a parthenogenetic gecko which has been increasingly expanding its range during the last century. This invasive species has been reported from multiple tropical and subtropical countries in five continents, most of which were colonized in recent times. In order to understand how the realized niche of the species was affected by this dramatic geographic range expansion, we reconstructed the history of the geographic range expansion. We built models of the realized niche of the species at different points in time during the invasion process. This was achieved through the implementation of modern hypervolume construction methods, based on the Hutchinson’s niche concept. The models were then compared to detect possible realized climatic niche expansion over time. Furthermore, we investigated possible pathways used by the species to spread. A progressive expansion of the realized niche was identified. As the species spread into new areas, we observed a tendency to colonize regions with warmer temperatures and higher precipitation rates. Finally, we found evidence for cargo shipping being the major pathway through which the species expands its range. Further studies on this topic should aim to investigate the role of biological interactions, and how they shape the distribution of *L*. *lugubris* on a global scale. A deeper understanding of this kind of processes will help us tackle the issue of invasive species, which has become a major challenge in conservation biology.

## Introduction

Biological invasions represent a phenomenon that has been growing rapidly during the last centuries. Depending on the level of invasion, an invasive species may affect the ecosystem and compete with native species for resources, altering biological interactions and the function of the ecosystem itself [1]. In order to prevent and deal with biological invasions under a changing climate, knowledge about invasion biology and the processes and traits that promote an invasion is needed, as well as about possible responses of alien species to the new environmental conditions [2].

The genus *Lepidodactylus* currently comprises 34 species of geckos native to Southeast Asia and the Pacific [3]. The majority of them are excellent trans-oceanic dispersers [4], but the most successful colonizer of the genus is undoubtedly the Mourning gecko *Lepidodactylus lugubris* [5]. *L*. *lugubris* is a nocturnal, parthenogenetic species occurring in tropical and subtropical regions, mainly in coastal habitats [6]. The species shows a high degree of clonal diversity [7]. Some clones occur more frequently than others. Clone 2NA seems to be a more efficient colonizer because of divergent ecological traits, which might be linked to the thermal biology, temporal and geographic distribution of the lineage [8]. However, evidence for a geographic predominance of a specific clone lineage based on genotype and physiological traits is still lacking.

A combined analysis of its karyotype and proteins has shown that *L*. *lugubris* is of hybrid origin and identified the Marshall Islands as the species most likely geographic origin [5, 7, 9]. It is difficult to properly delineate a native range for the species, as early colonization events in the pacific islands and in the Asian continent have not been well documented during the first stages of the geographic range expansion. However, an increasing number of more recent geographic range expansions has been efficiently reported during the last years.

Occurrences of *L*. *lugubris* have been confirmed on numerous tropical and subtropical islands in the Pacific, as well as in Southeast Asia, Australia, Papua New Guinea, and several islands in the Indian Ocean. During the last decades, the species has also reached several Central and South American countries, and it is continuing to spread [6]. Recently, the species has been reported from Thailand [10] and has established populations in the Caribbean [11, 12, 13, 14, 15, 16, 17, 18].

Whether a species should be considered invasive or just alien in a new ecosystem may depend on different definitions and theoretical backgrounds. We refer to *L*. *lugubris* as an invasive species according to the definition given by [19]. A biological invasion is defined as a human-mediated introduction of a species, which establishes vital populations and spreads well beyond its potential range, as delimited by their natural dispersal mechanisms and biogeographic barriers. Data concerning the ecological impact on native species and economic consequences of *L*. *lugubris* introductions is currently lacking.

In invasion biology research, it is often assumed that the realized niche of a species is conservative, and therefore it does not vary in the invasive range [20, 21, 22]. However, a number of studies provided evidence that this is not always true. Changes in the realized niche have been detected in both plants [23, 24] and animals [25, 26, 27], revealing some degree of plasticity in the realized niche of the species. Currently, knowledge about realized niche expansions in geckos is restricted to studies on *Tarentola mauritanica* [28] and *Hemidactylus turcicus* [29]. *L*. *lugubris* is known to be quite flexible when it comes to thermoregulation. Its body temperature in a tropical habitat such as Hawaii can range between 25°C and 35°C at different times of the day, reaching lower temperatures than those of other species of gecko such as *H*. *frenatus* in the same area [30]. In general, the species is able to adapt to a wide range of temperatures in its habitat [31, 32]. Physiological data on the species therefore already suggests a possible plasticity of the realized niche which could facilitate the species in the process of colonizing new areas. For instance, when the area is characterized by different environmental conditions such as a different minimum and maximum temperature.

**Fig 1:**
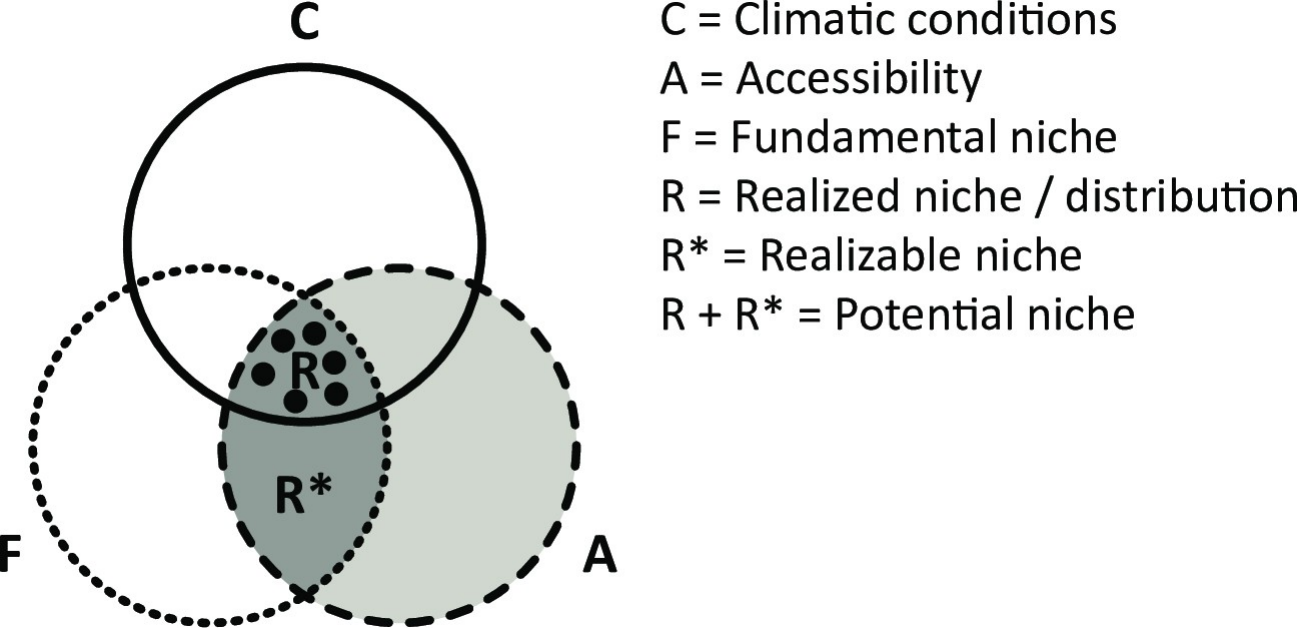
The diagram clarifies the concept of realized niche which was adopted in this study. The realized niche is represented by the intersection of three major factors influencing the distribution of a species: Climatic conditions, Accessibility and Fundamental niche. The realized niche is a subset of the potential niche of the species, it refers to the environmental space currently used by the species in the areas where it is distributed. The realizable niche represents the existing environmental space which the species is initially not able to explore due to lack of accessibility (e.g. abiotic and biotic barriers).

Our hypothesis in this study refers to the realized niche of the species, as illustrated in Fig 1. As the species was able to expand its geographic range so extensively across the globe, we expected the realized niche of the species to be affected by the geographic range expansion. Major changes in the geographic distribution of the species should be reflected in environmental space as well. Referring to the diagram in Fig 1, we expected the species to explore new areas of its potential niche. This assumes that the realized niche can expand and fill additional space of the realizable niche in environmental space due to an increase in accessible geographic area. A secondary purpose of the study was to investigate how *L*. *lugubris* has spread so successfully and what is the most likely pathway used by the species to colonize new areas.

Based on the Hutchinson’s niche concept, we implement a modern hypervolume construction method [33, 34] in order to capture the realized niche of *L*. *lugubris* at different stages of the invasion process. We then compared the niche hypervolumes and analyzed changes in the shape of the hypervolumes over time.

## Materials and methods

### Range expansion reconstruction

A total of 1106 species records reported between 1891 and 2017 were included in the occurrence dataset (S1 Dataset). The records cover the whole currently known distribution of the species worldwide. The dataset was assembled with 1038 records obtained from the Global Biodiversity Information Facility (GBIF.org 29th February 2016), 14 were obtained from specimens belonging to the collection of the Zoological Research Museum Alexander Koenig in Bonn. The remaining 54 were obtained from scientific literature [6, 13, 35, 36, 37, 38, 39, 40, 41]. Erroneous and duplicate records were excluded from the dataset using the R package CoordinateCleaner version 2.0–11. We discarded records for which the coordinates were located out of the land boundaries. We also removed records located in areas which are to be considered extremely unsuitable for the species due to the environmental conditions and for which no reliable source of information reporting the presence of the species in those areas was available.

To analyze the geographic range expansion over time, the occurrence data was divided into eight subsets: the first subset contains all species records from 1890 to 1950, with a total of 75 records across the whole geographic range of the species, as it was known until 1950. Starting with a 60 years record subset allows us to build a first niche model with a solid record base, which prevents us from excluding important information about the initial shape of the species realized climatic niche. As previously mentioned, we are unable to identify a true native range for the species. However, by including records from a 60 years span, we can be confident that the first niche model will enclose the shape of the species niche within its native range. This means that changes of the shape of the realized niche in the environmental space which will be detected in the subsequent hypervolumes should be linked to the geographic range expansion. Moreover, hypervolumes require a minimum number of records to be computed. In high dimensional hyperspace, having a relatively high number of species records affects the hypervolume making it more accurate.

In the subsequent steps, we cumulatively added the records for each decade until the final subset, which contains all the records collected to date. A geographic range expansion map was built by plotting the species’ presence points on a map in QGIS; the 1:10m resolution map was obtained from Natural Earth (http://www.naturalearthdata.com).

### Hypervolumes and niche modelling

The environmental predictors were obtained from WorldClim–Global Climate Data, version 2.0 [42] in the form of 19 bioclimatic variables (Table 1), with a resolution of 2.5 arc minutes. The variables are derived from temperature and precipitation, two environmental factors which are considered important drivers of distribution for an ectotherm organism occurring in tropical areas. Temperature also affects the reproductive biology [43], as it happens for many reptiles. Based on these considerations, the 19 bioclimatic variables have a high potential of capturing the climatic realized niche of the species in the environmental space. In version 2.0, the variables are related to a combination of remote sensing data and interpolated climatic station data [42], making it much more accurate for oceanic islands and areas with a low station density. Because the global analysis implied high computational costs, predictors were masked by an overall climatic envelope model (BIOCLIM) which was trained using the full set of species records to reduce projection space. By definition, the maximum possible hypervolume model is enclosed in the BIOCLIM model representing a multidimensional rectangular box car envelope.

**Table 1:** Summary of the principal component analysis showing Pearson's correlation coefficients for each predictor, eigenvalues and explained total variance.

Variable	PC1	PC2	PC3	PC4
Annual Mean Temperature	0.863	0.498	0.006	0.048
Mean Diurnal Range	-0.232	-0.306	-0.113	0.881
Isothermality	0.783	-0.164	-0.411	0.278
Temperature Seasonality	-0.848	0.002	0.384	-0.126
Max Temperature of Warmest Month	0.635	0.579	0.175	0.384
Min Temperature of Coldest Month	0.909	0.371	-0.125	-0.106
Temperature Annual Range	-0.801	-0.107	0.291	0.409
Mean Temperature of Wettest Quarter	0.710	0.521	0.310	-0.106
Mean Temperature of Driest Quarter	0.824	0.354	-0.279	0.167
Mean Temperature of Warmest Quarter	0.683	0.650	0.204	-0.009
Mean Temperature of Coldest Quarter	0.917	0.361	-0.133	0.070
Annual Precipitation	0.832	-0.500	0.212	0.027
Precipitation of Wettest Month	0.693	-0.330	0.547	0.144
Precipitation of Driest Month	0.780	-0.532	-0.056	-0.136
Precipitation Seasonality	-0.477	0.506	0.492	0.250
Precipitation of Wettest Quarter	0.727	-0.319	0.532	0.124
Precipitation of Driest Quarter	0.762	-0.572	-0.067	-0.110
Precipitation of Warmest Quarter	0.612	-0.410	0.494	-0.227
Precipitation of Coldest Quarter	0.636	-0.594	-0.109	0.210
**Eigenvalue**	10.390	3.649	1.827	1.467
**Explained Variance**	54.682	19.208	9.614	7.719

For a correct delineation of the hypervolume shape with the chosen algorithm, an orthogonal space is required. Therefore, a Principal Component Analysis (PCA) was performed on the original 19 bioclimatic variables with the stats package in Cran R (R Core Team, 2018). Principal components (PCs) with an eigenvalue >1 were used as predictors for the calculation of the hypervolumes.

As algorithm to build the hypervolumes we used one-class support vector machines (SVM), available in the hypervolume package [44]. SVM is a one-classification machine learning method that selects a subset of points classified as support vectors; it uses positive observations of data to identify regions of the hyperspace and classify them as either 1 or 0, in or out of the hypervolume, respectively. The SVM is implemented with a radial basis function (RBF). Only two parameters can be tuned by the user. The **γ** parameter controls the width of the RBF function, the ν parameter determines the upper and lower bound on the fraction of misclassification errors. We set the parameters as γ = 0.5 and ν = 0.01. These are the standard parameters for the algorithm. The SVM is particularly suited for our study because it aims at identifying the true boundaries of a species’ niche. All hypervolumes were projected into hyperspace and, subsequently, into geographic space using the “hypervolume_project” function of the hypervolume package in Cran R. The complete R script is available in “S1 Text”.

The model performance was evaluated by the True Skills Statistic (TSS) [45], the same procedure was applied to each SDM. The following steps were performed in QGIS. First, a minimum convex polygon (MCP) was computed enclosing the species occurrence data which were used previously to build the respective model, centered to the expected native range. We used the MCP to clip the geographic space where the species is occurring and generated 5000 random records across the clipped area for hypervolume “2010s”, which contains all the species records reported until 2017. For the remaining seven hypervolumes, we generated a number of random records that was proportionally decreasing together with the actual number of species records, and therefore with the size of the MCP used to clip the geographic space. In doing so, we ensured that the evaluation of the performance is comparable between the models. Finally, an confusion matrix was generated by counting the number of cells for which presence was correctly predicted by the model, number of cells for which the species was not found but the model predicted presence, number of cells for which the species was found but the model predicted absence and number of cells for which absence was correctly predicted by the model. These four scores represent the input data for the TSS formula (S1 Appendix).

Pairwise overlap statistics of the hypervolumes were performed by means of Jaccard similarity and Sorensen similarity computed with the relevant functions of the Hypervolume package. Frequency distributions of each subset of records against each bioclimatic variable, and secondarily against each principal component, were plotted. All statistical tests, frequency plots, and calculations of area in geographic space were performed in Cran R.

## Results

### Geographic range expansion

**Fig 2:**
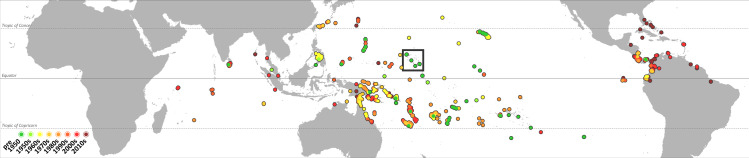
*Lepidodactylus lugubris* has been expanding its range during the last decades. The species’ current range is marked by a black margin. Dots represent occurrence records and are colored according to the period they were first recorded in. The square marks the species’ geographic origin in the Pacific.

The oldest records of *L*. *lugubris* are found mainly in the Pacific region and coincide with the Marshall Islands and the adjacent Tarawa atoll (Cook Island) (Fig 2). The highest density of recent records, and at the same time low densities of old records, is found in South and Central America, Australia and New Guinea, and islands in the Indian Ocean. The geographic range expansion varies regionally between the examined time slices. The first major expansion can be observed in the 1960s in the Philippines and in Indo-Australia. Further records from Panama and novel records from Ecuador were also documented in this period, although still scarce. Except for the Philippines, the species then continuously spread in these invaded regions, especially in the Americas, while at the same time the species had colonized the Indo-Malayan region and spread further westward across the Indian Ocean. In the last decade, *L*. *lugubris* again extended its range mainly in the Americas, most noteworthy entering the Caribbean and moving east of the Andes.

### Environmental predictors and PCA

The PCA performed on the original environmental predictors resulted in four principal components with eigenvalues > 1 (Table 1). The four PCs were used as predictors to build the hypervolumes. PC1 shows highest correlation with “Mean Temperature of Coldest Quarter” (0.917), “Mean Temperature of Coldest Month” (0.909), and “Annual Mean Temperature” (0.863); it represents 55% of the total variance. PC2 is mostly correlated with “Mean Temperature of Warmest Quarter” (0.650), “Precipitation of Coldest Quarter” (0.594), and “Max Temperature of Warmest Month” (0.579), and it represents 19% of the total variance. PC3 is mostly correlated with “Precipitation of Wettest Month” (0.547), PC4 with “Mean Diurnal Range” (0.881) and “Temperature Annual Range” (0.409). These last two PCs explain 10% and 8% of the total variance, respectively.

### Hypervolumes of bioclimatic niches and overlap statistics

**Fig 3:**
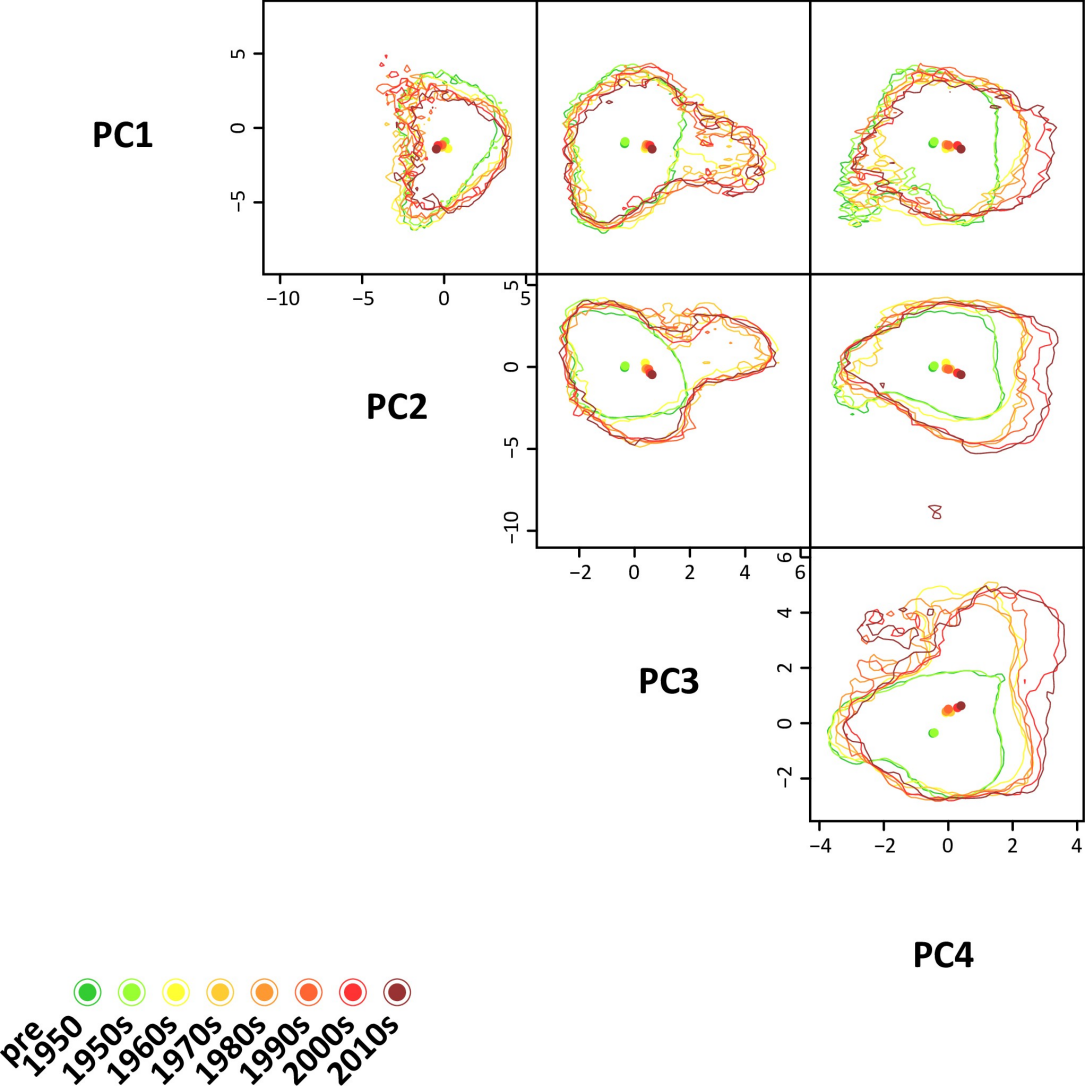
Changes in the four-dimensional hypervolumes of the realized bioclimatic niche of *Lepidodactylus lugubris* over time. Dots represent the centroids of each hypervolume.

A total of eight hypervolumes were obtained and projected into hyperspace (Fig 3) using the subsets of data which were generated in the data preparation phase. Each hypervolume represents a niche model based on one of the eight nested subsets characterizing the realized niche volume of the species at a specific point in time of the invasion history. Observing the outlines of the hypervolumes, a progressive niche expansion through time in the 2^nd^, 3^rd^ and 4^th^ dimensions, which correspond to PC2, PC3 and PC4, is clearly detectable. The centroids of the hypervolumes indicate the direction of the expansion. The extent of the expansion over time can be measured in terms of PC units: in PC2 and PC4 an expansion of 2 PC units is detected, while the strongest expansion is observed in PC3, which shows a total extent of 5 PC units. Although the extent of the expansion progressively grows over time, in PC2 and PC3 it becomes much more prominent between the hypervolumes for the 1950s and the 1960s. It appears that within these periods the realized niche expanded rapidly and to a greater extent in the PC2 and PC3 dimensions.

In both the Jaccard and Sorensen index, the overlap score decreases with increasing temporal distance between compared hypervolumes, i.e. hypervolumes based on old records show the least overlap with hypervolumes that include also recent data (Table 2). Portions of hyperspace which are included in more recent hypervolumes were previously not included in the older ones, suggesting that novel areas of the hyperspace have been explored over time. The most conspicuous drop in the overlap scores is found between the hypervolumes for the 1950s and the 1960s. For both Jaccard index and Sorensen index, the drop extent is about 0.9. These results are consistent with what was observed in the projections of the hypervolumes into both hyper- and geographic space. A decrease in overlap is correlated to the increased number of records; an increase of the geographic range corresponds to an increase of the realized niche. So, a decrease in overlap must be interpreted as an extension of the realized niche.

**Table 2:** Overlap statistics by means of Jaccard (above diagonal) and Sorensen (below diagonal) indices. Both indices suggest a progressive decrease of overlap, as more distant hypervolumes are compared. Area (in km^2^) covered by each hypervolume when projected in geographic space is increasing over time.

	pre 1950	1950s	1960s	1970s	1980s	1990s	2000s	2010s
**pre 1950**		0.903	0.481	0.367	0.321	0.290	0.267	0.227
**1950s**	0.898		0.475	0.359	0.315	0.285	0.262	0.224
**1960s**	0.846	0.912		0.701	0.595	0.536	0.483	0.423
**1970s**	0.791	0.857	0.920		0.797	0.716	0.637	0.556
**1980s**	0.715	0.778	0.834	0.887		0.851	0.751	0.654
**1990s**	0.595	0.652	0.698	0.746	0.824		0.838	0.733
**2000s**	0.366	0.416	0.444	0.479	0.528	0.644		0.816
**2010s**	0.370	0.421	0.450	0.486	0.536	0.649	0.949	
**Area [km**^**2**^**]**	5,558,793	6,138,923	10,046,385	12,348,408	13,531,020	13,649,181	14,621,780	15,231,322

### Frequency distribution of climatic variables

**Fig 4:**
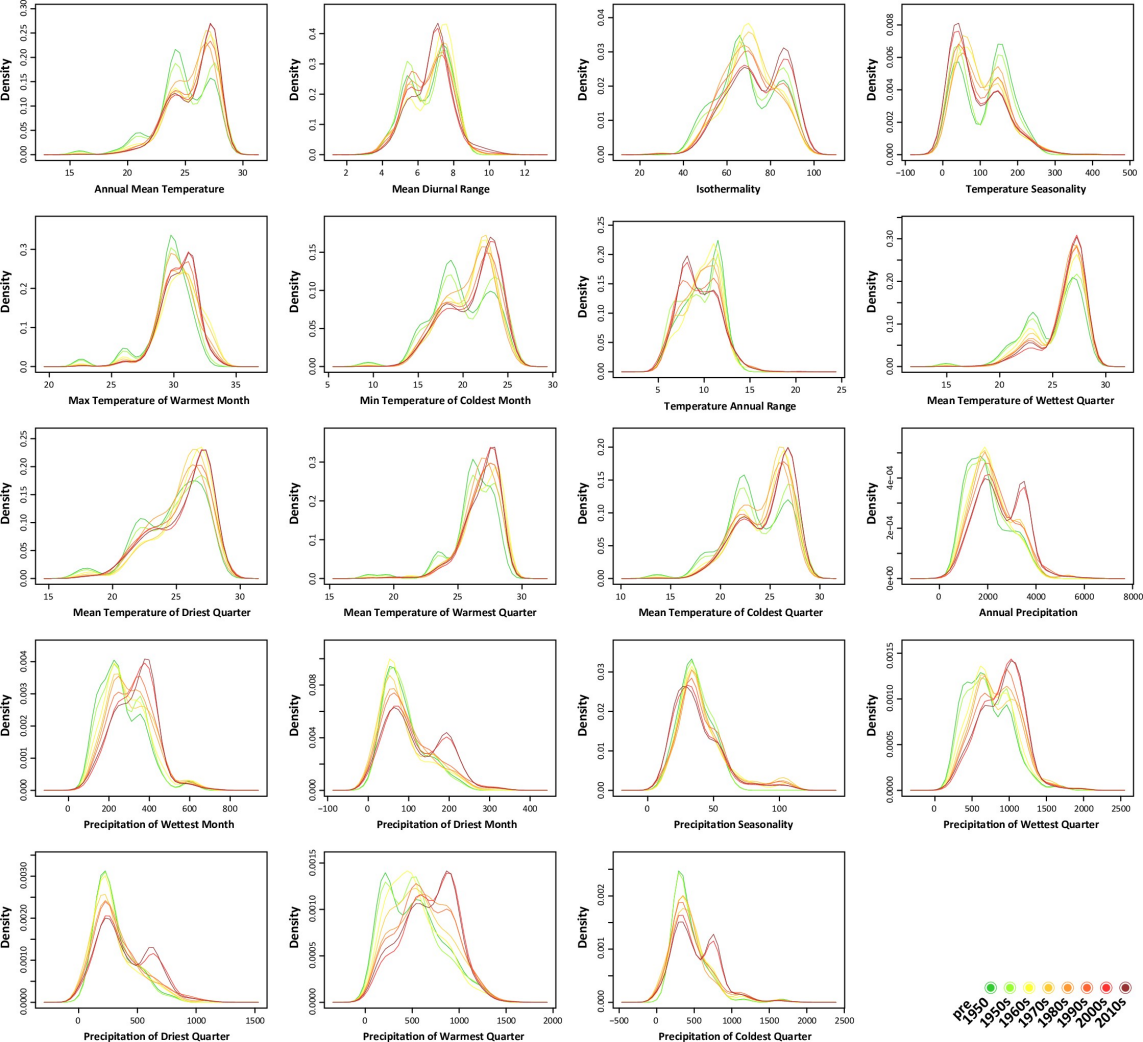
Density distributions of the 19 bioclimatic variables for each period.

Density distributions of each subset of records for each of the 19 bioclimatic variables (Fig 4) provide a more detailed assessment on climatic niche expansion that the species has experienced during its invasion history. We consider a realized niche expansion when the ranges of the density distributions increase their extent in more recent niche hypervolumes. In other words, when novel environmental conditions that have not been covered previously are now included in the density distribution range. A major expansion is detectable for “Max Temperature of Warmest Month” (Fig 4). Minor expansions can be found for “Annual Precipitation” and “Mean Temperature of Warmest Quarter”. During the last decades, the species has expanded its thermal range towards warmer temperatures (e.g. expansion in “Max Temperature of Warmest Month” and “Mean Temperature of Warmest Quarter”). Furthermore, the distribution of “Annual Precipitation” suggests a geographic range expansion into areas with a higher precipitation.

For the other climatic variables, a change in the position of the peaks can be observed in most of them. In these cases, the species does not expand into novel conditions, but the position of the peaks shifts within the already realized niche. The density of records for the species is increasing in areas with warmer temperatures (e.g. “Annual Mean Temperature” and “Min Temperature of Coldest Month”) and higher precipitation (e.g. “Precipitation of Wettest Month” and “Precipitation of Warmest Quarter”). These trends fit the pattern in the niche expansion described above. In addition, an increase in density of records in areas with smaller temperature range (“Temperature Annual Range”) can be observed.

**Fig 5:**
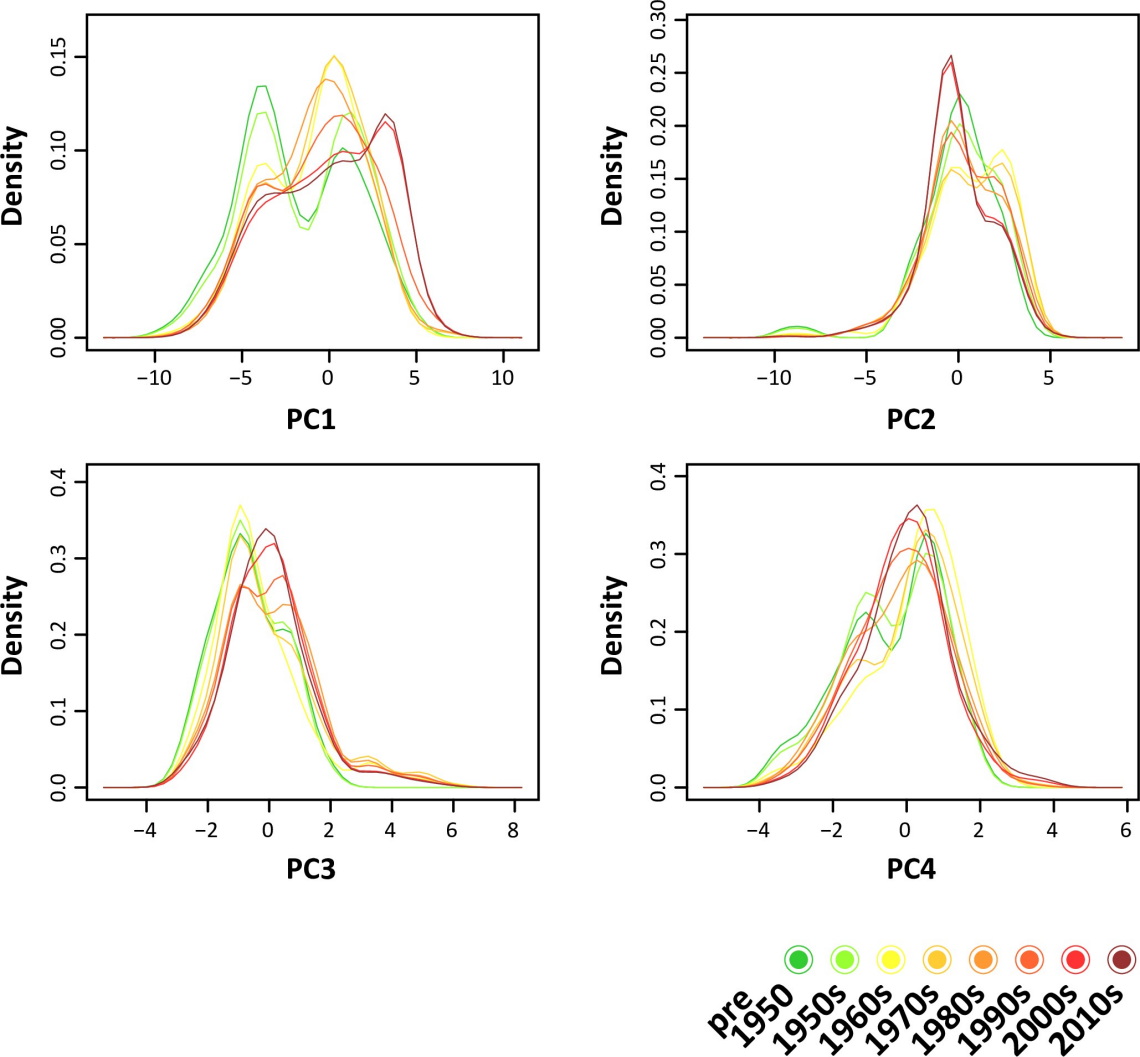
Density distributions of the four principal components (PCs) for each period.

In the density distributions of the PCs (Fig 5), PC1 shows a progressive and clearly visible niche expansion: while the ranges almost overlap pre-1950 and in the 1950s, the subsequent density distributions gradually detach from these. The same pattern can be observed in PC3, although not as pronounced as in PC1. No clear niche expansion could be detected in PC2 and PC4.

### Species distribution models

**Fig 6:**
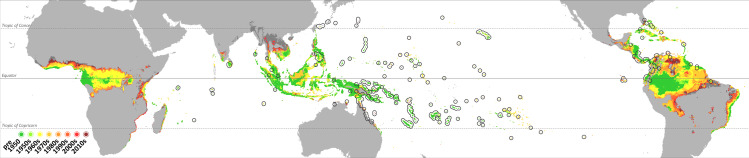
Species distribution models of *Lepidodactylus lugubris* for different periods predict a change in suitable areas over time. SDMs were obtained by projecting the four-dimensional hypervolumes of the realized bioclimatic niches (Fig 3) into geographic space. Dark grey areas show the projection mask generated using an envelope model and black margins encompass the current range of the species. See S1 Fig for single SDMs.

Each hypervolume is projected into geographic space in Fig 6. The projection highlights areas which are considered suitable for the species, according to our niche model, mainly: Central Africa, African South-Eastern coast, Madagascar, South India, Southeast Asia, Australian North-Eastern coast, all of the Pacific islands, Tropical America (Including Central America, Tropical South-America and the Caribbean). The total suitable area visually expands over time. The geographic extent of the expansion is reported in Table 2, as increasing km^**2**^ of suitable area over time. The distribution model largely matches the currently known distribution of *L*. *lugubris*. However, our model also detected large areas in tropical Africa and Amazonia as potentially suitable for *L*. *lugubris*, although no records from these regions have been reported so far. Approximately 5% of the species records from North-East Australia and Florida were left out of the hypervolume by the algorithm due to the thresholding procedure. In high dimensional hyperspace, records which are lying on the borders of the hypervolume due to their extreme conditions in the climatic space can be left out of the delineation process.

The TSS scores for the eight SDMs ranged between 0.6 and 0.7. Six of them scored 6.0 (pre1950s, 1950s, 1960s, 1970s, 1980s and 1990s) and two of them scored 0.7 (2000s and 2010s). Error matrices used for the TSS and detailed results of the test can be found in Supplementary materials (S1 Text, S1 Appendix, S1 Fig).

## Discussion

### Pathways of invasion

Invasive lizards are known to spread mainly through two pathways: cargo shipping and pet trade [46, p.35-37]). It has been suggested that *L*. *lugubris* may also be spreading through cargo shipping [47]. The characteristics of its egg shell would facilitate the use of this pathway, as the rigid shell is resistant to desiccation and poorly permeable to salt water [48].

*Lepidodactylus lugubris* is also a common species in the American and European pet trade market [49]. Thus, pet trade could be also playing a role in *L*. *lugubris* range expansion, for instance in Florida, where the connection between pet trade and many reptile invasions is well known [50]. However, a comparison between the range expansion map (Fig 2) and a map of the main maritime bottlenecks and shipping routes reported in “The Geography of Transport Systems” [51, p.31], suggests that the main pathway used by *L*. *lugubris* to spread all over the globe might be cargo shipping. A web of both Core and Secondary routes connects areas in which *L*. *lugubris* occurs across the whole geographic range.

The first records of *L*. *lugubris* ever reported from the Americas came from Panama, in the early 20^th^ century. The canal was officially opened in 1914, just two years before *L*. *lugubris* presence in Panama was registered, indicating Panama as the most likely epicenter of *L*. *lugubris* invasion in the new world. Interestingly, no Core or Secondary route directly connects the Americas or Asia to Central Africa. The SDM obtained by the projection of the hypervolume identifies a large area of suitable climatic conditions for *L*. *lugubris* in Central Africa. However, no records have been reported to date from the African mainland. The lack of linking pathways such as shipping routes can explain this circumstance.

Biological interactions might also be playing a role in shaping the geographic distribution of *L*. *lugubris*. Examples of competition for resources are known for *Hemidactylus frenatus*, of which the range largely overlaps with that of *L*. *lugubris* [52], and within this range, *H*. *frenatus* could potentially outcompete *L*. *lugubris* for resources [53, 54]

### Niche expansion and SDMs

The progressive expansion of the hypervolumes of the niche of *L*. *lugubris* can be observed in at least two dimensions, namely PC 3 and PC 4. PC 2 was mostly correlated with “Mean Temperature of Warmest Quarter”, “Max Temperature of Warmest Month” and “Precipitation of Wettest Month”. PC 3 with “Precipitation of Wettest Month” and “Precipitation of Warmest Month”. PC 4 with “Mean Diurnal Range and “Temperature Annual Range”. These results suggest that the species has been expanding its realized niche in directions that are linked to warmer temperatures and a higher variation in precipitation, as well as with the temperature range of its habitat. This is also corroborated by the results of the univariate analyses (Fig 4).

Although details about the niche expansion are difficult to obtain from looking solely at the hypervolumes, some changes affected the shape of the realized niche of *L*. *lugubris* through time. The hypervolumes in Fig 3 show that the niche of the species is expanding along specific axes which are mostly correlated with specific bioclimatic variables. Additionally, the increasing size of the hypervolume over time is confirmed by the overlap statistics, for which the results are displayed in Table 2.

Clear density distribution range expansions are noticeable in the density plots (Fig 4) of three climatic variables, namely in “Max Temperature of Warmest Month”, “Mean Temperature of Warmest Quarter” and “Annual Precipitation”. In all three variables, the range is shifting towards higher values of the variables. This indicates that the species, over the years, has expanded the geographic range in areas that reach warmer temperatures during the warmest months; these temperatures were not originally present within the habitat’s temperature range of the species. Moreover, *L*. *lugubris* has colonized areas with a higher annual precipitation than those in which it originally occurred.

Apart from the niche expansions mentioned above, important changes in the position of the peaks are observed in the density distribution of five distinct variables: “Annual Mean Temperature”, “Min Temperature of Coldest Month”, “Precipitation of Wettest Month”, “Precipitation of Warmest Quarter” and “Temperature Annual Range”. In the first four variables, the position of the peak shifted towards higher values.

A growing peak of the curve represents an increasing number of records of *L*. *lugubris* coming from areas, within the species distribution, which match a specific value of a specific variable (e.g. “Annual Mean Temperature” = 25°C). If we assume that an increasing number of records might be linked to an increasing population density of the species in certain areas, the interpretation of these results would be that the population density of the species is increasing in areas which present warmer temperatures and a higher precipitation rate. Further, the peak of the “Temperature Annual Range” shifted towards lower values; thus, the population density might have increased in areas with a less pronounced temperature variation throughout the year. The trends observed in the distribution density plot are consistent with those observed in the hypervolume expansion: the species shows a tendency to colonize and increase its population density in warmer areas, with higher precipitation rate and a smaller temperature range.

The eggs of *Lepidodactylus lugubris* have been proven to hatch even with an external temperature of 14°C; while eggs of *H*. *frenatus* would not hatch below 20°C [43]. Our data suggest that the annual mean temperature of the areas in which *L*. *lugubris* occurs, ranges approximately between 19°C and 29°C (Fig 4). The minimum temperature of the coldest month is around 14°C; this suggests that the species is not occurring in areas that reach temperatures which do not allow the eggs to hatch.

In many reptiles, temperature of incubation influences hatchlings phenotype such as sex, size, shape and locomotive performance [55, 56]. Higher incubation temperatures have been found to accelerate growth and increase size of the hatchlings in *Alligator mississippiensis* [57]. Gravid females of *L*. *lugubris* thermoregulate their body temperature at higher temperatures than other gekkonid species [32]. Therefore, warmer temperatures might facilitate the colonization of new areas by positively affecting the development of the hatchlings in the eggs.

The projection of the hypervolumes into geographic space (Fig 6) reveals an increasing suitable area predicted by the model, as more recent records are considered. This means that the niche expansion can be visualized and measured also geographically, directly on the world map, as new areas become suitable to the species. Overall, the realized niche model included all the areas in which the species occurs, as expected, and identified large tropical and subtropical areas as suitable for the species. As the algorithm excludes a small percentage of the records which are located on the border of the hypervolume based on the automatically generated threshold, some of the points which occurred in areas with environmental conditions that differ the most from the average suitable climate for the species were excluded from the hypervolume. This is the case for some of the occurrence records in Australia and Florida. Even so, we detected a substantial expansion of the realized niche over time.

The TSS scores for the eight SDMs ranged between 0.6 and 0.7 (S1 Appendix). This is considered a fair performance for an SDM based on the TSS [58]. The overall performance was precise, even in very small areas such as the Hawaii islands. However, the main goal of this study was not to build a predictive model that could precisely highlight potential suitable areas, but to build a model that could detect changes in the realized niche of the species based on its known distribution. Thus, a more cautious consideration of the extreme records in our dataset does not affect the robustness of our results.

## Conclusion

In conclusion, we found that *L*. *lugubris* is tending to increase its population and extending its geographic range in areas which present a warmer climate and with an increased precipitation. The geographic range expansion is reflected in a realized niche expansion in environmental space. These tendencies might enhance a further expansion of the range under a global warming, as new areas might become accessible to the species. In addition, we reconstructed the history of its range expansion over time, and identified possible pathways, such as cargo shipping, used by the species to increase its distribution. A consequent step from here would be to better understand the role of biological interactions and include them as a dimension that shapes the niche of the species.

## Supporting information

S1 Dataset (CSV)

S1 Fig Animated map of the species distribution models. Chronological sequence of the realized bioclimatic niche of *Lepidodactylus lugubris* projected into geographic space. The black circles show the occurrence records for each period and are added cumulatively.
(GIF)

S1 Text Hypervolume R Script. R code used to build the hypervolumes.
(TXT)

S1 Appendix True skill statistics results. A summary of the confusion matrices used for the TSS and the TSS results for each SDM.
(PDF)
