# Supplementary figures and images for: Continuous expansion of the geographic range linked to realized niche expansion in the invasive Mourning gecko Lepidodactylus lugubris (Duméril & Bibron, 1836)
Source: PLoS One. 2020 Jul 6;15(7):e0235060. doi: 10.1371/journal.pone.0235060 (PMC7337341; doi:10.1371/journal.pone.0235060)

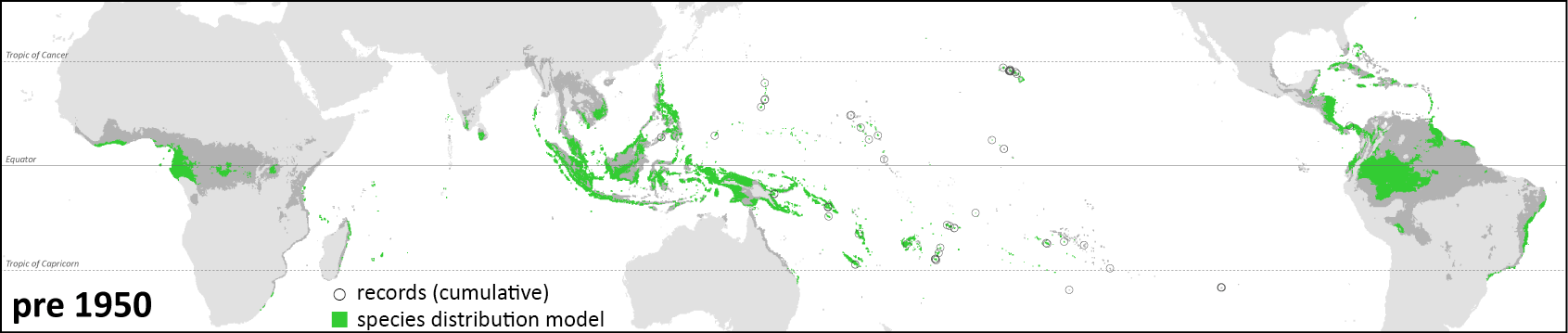

Supplement: S1 Fig — Chronological sequence of the realized bioclimatic niche of Lepidodactylus lugubris projected into geographic space. The black circles show the occurrence records for each period and are added cumulatively. (GIF) [file pone.0235060.s002.gif]
