# Supplementary material for: Continuous expansion of the geographic range linked to realized niche expansion in the invasive Mourning gecko Lepidodactylus lugubris (Duméril & Bibron, 1836)
Source: PLoS One. 2020 Jul 6;15(7):e0235060. doi: 10.1371/journal.pone.0235060 (PMC7337341; doi:10.1371/journal.pone.0235060)
Supplement: S1 Appendix — A summary of the confusion matrices used for the TSS and the TSS results for each SDM. (PDF) [file pone.0235060.s004.pdf]

Confusion matrices and True Skill Statistics scores for the SDM evaluation

Hypervolume pre-1950s

		Validation dataset	
		Presence	Absence
Model	Presence	64	227
	Absence	8	98

Total Random points = 325

TSS = 0.6

Hypervolume 1950s

		Validation dataset	
		Presence	Absence
Model	Presence	69	307
	Absence	17	82

Total Random points = 389

TSS = 0.6

Hypervolume 1960s

		Validation dataset	
		Presence	Absence
Model	Presence	180	634
	Absence	8	216

Total Random points = 850

TSS = 0.6

Hypervolume 1970s

		Validation dataset	
		Presence	Absence
Model	Presence	270	898
	Absence	14	386

Total Random points = 1284

TSS = 0.6

Hypervolume 1980s

		Validation dataset	
		Presence	Absence
Model	Presence	405	1375
	Absence	22	555

Total Random points = 1930

TSS = 0.6

Hypervolume 1990s

		Validation dataset	
		Presence	Absence
Model	Presence	566	1850
	Absence	27	831

Total Random points = 2681

TSS = 0.6

Hypervolume 2000s

		Validation dataset	
		Presence	Absence
Model	Presence	862	3001
	Absence	26	1013

Total Random points = 4014

TSS = 0.7

Hypervolume 2010s

		Validation dataset	
		Presence	Absence
Model	Presence	1063	3421
	Absence	43	1579

Total Random points = 5000

TSS = 0.7
